# Effects of Scene Properties and Emotional Valence on Brain Activations: A Fixation-Related fMRI Study

**DOI:** 10.3389/fnhum.2017.00429

**Published:** 2017-08-31

**Authors:** Michał Kuniecki, Kinga B. Wołoszyn, Aleksandra Domagalik, Joanna Pilarczyk

**Affiliations:** ^1^Psychophysiology Laboratory, Institute of Psychology, Jagiellonian University Kraków, Poland; ^2^Neuroimaging Research Group, The Małopolska Centre of Biotechnology, Jagiellonian University Kraków, Poland

**Keywords:** fMRI, eye movements, attention, visual noise, object, scene perception

## Abstract

Temporal and spatial characteristics of fixations are affected by image properties, including high-level scene characteristics, such as object-background composition, and low-level physical characteristics, such as image clarity. The influence of these factors is modulated by the emotional content of an image. Here, we aimed to establish whether brain correlates of fixations reflect these modulatory effects. To this end, we simultaneously scanned participants and measured their eye movements, while presenting negative and neutral images in various image clarity conditions, with controlled object-background composition. The fMRI data were analyzed using a novel fixation-based event-related (FIBER) method, which allows the tracking of brain activity linked to individual fixations. The results revealed that fixating an emotional object was linked to greater deactivation in the right lingual gyrus than fixating the background of an emotional image, while no difference between object and background was found for neutral images. We suggest that deactivation in the lingual gyrus might be linked to inhibition of saccade execution. This was supported by fixation duration results, which showed that in the negative condition, fixations falling on the object were longer than those falling on the background. Furthermore, increase in the image clarity was correlated with fixation-related activity within the lateral occipital complex, the structure linked to object recognition. This correlation was significantly stronger for negative images, presumably due to greater deployment of attention towards emotional objects. Our eye-tracking results are in line with these observations, showing that the chance of fixating an object rose faster for negative images over neutral ones as the level of noise decreased. Overall, our study demonstrated that emotional value of an image changes the way that low and high-level scene properties affect the characteristics of fixations. The fixation-related brain activity is affected by the low-level scene properties and this impact differs between negative and neutral images. The high-level scene properties also affect brain correlates of fixations, but only in the case of the negative images.

## Introduction

Visual information acquisition is not a continuous process. It is partitioned into fixations, during which visual information is extracted. Portions of information acquired during fixations may be regarded as “units of information” (Marsman et al., 2012), the processing of which can be systematically analyzed, providing insight into perceptual and cognitive mechanisms of vision, including attention. The major function of attention is to filter the most important information. Indeed, in free viewing conditions spatial deployment of attention is reflected in the temporal and spatial characteristics of fixations (for a review see Henderson, 2003). Specifically, fixations are not randomly distributed; their location and duration strongly depend on the informative value linked to both high-level scene characteristics, such as object-background composition, and basic low-level physical properties, such as signal-to-noise ratio (Buswell, 1935; Mackworth and Morandi, 1967; Yarbus, 1967; Kayser et al., 2006; Henderson et al., 2009; Ossandón et al., 2012; Glaholt et al., 2013; Henderson et al., 2014; Onat et al., 2014). Interestingly, emotional content strongly attracts attention and influences eye movements, possibly due to its evolutionary and behavioral relevance (Calvo and Lang, 2004; Nummenmaa et al., 2006; Calvo et al., 2007, 2008; Humphrey et al., 2012; Niu et al., 2012; Kaspar et al., 2013; Pilarczyk and Kuniecki, 2014 ). Moreover, it has been shown that the emotional content modulates the impact of both high and low-level features of a scene on the processing of visual information and attentional deployment (Humphrey et al., 2012; Todd et al., 2012b; Pilarczyk and Kuniecki, 2014). In the present study, we aimed to examine the brain underpinnings of this modulatory effect using the recently developed fixation-based event-related (FIBER) method of fMRI data analysis (Marsman et al., 2012).

One of the essential components of vision is the detection and identification of objects. It is a process requiring scene decomposition into discrete objects and background (Henderson and Hollingworth, 1999). Various lines of research show that emotional objects are easily detected and preferentially capture attention (Ohman et al., 2001; Nummenmaa et al., 2006; Humphrey et al., 2012; Niu et al., 2012; McSorley and van Reekum, 2013; Pilarczyk and Kuniecki, 2014). Eye-tracking studies have shown that while viewing emotional scenes, attention is instantly drawn to objects rather than the background, whereas objects in neutral scenes do not attract attention to the same extent (Humphrey et al., 2012; Niu et al., 2012; Pilarczyk and Kuniecki, 2014). Furthermore, the interaction between emotional content and object-background composition has also been shown in the EEG studies, using the steady state visual evoked potentials (ssVEPs) and event-related potentials (ERP). Emotional images, as a whole, evoke greater brain activations, measured as the magnitude of the ssVEPs, than neutral ones (Keil et al., 2003). However, if the attentional focus is cued towards the background of an emotional picture, the ssVEP response is indistinguishable from the ssVEP response to a neutral object (Hajcak et al., 2013). Only after focusing spatial attention onto the key emotional object, the enhancement of the ssVEP and late positive potential, an ERP component linked to emotional processing, does emerge (Keil et al., 2005; Hajcak et al., 2013). This effect is further supported by Ferri et al.’s (2013) fMRI study, which showed that focusing overt attention on an emotional region within a negative image, compared to a non-emotional region, is linked to greater activity not only in brain areas related to emotion, such as the amygdala and insula, but also in the part of the visual cortex, inferior occipital gyrus. This pattern of results demonstrates that fixating key emotional objects, indeed, changes brain activity, as compared to fixating both a neutral object and the background of an emotional scene. Presumably, this change of brain activity occurs due to enhanced processing of emotional visual information (Lang et al., 1998; Bradley et al., 2003; Junghöfer et al., 2006; Todd et al., 2012a).

Regarding the low-level properties of stimuli, superimposing visual noise over an image affects its informative value on a more basic level, by limiting the availability of visual information. Visual noise influences the activity of the visual cortex, however, only within the higher visual areas linked to object recognition and scene comprehension, such as the V4, lateral occipital cortex (LOC), fusiform face area and mid-fusiform area (Grill-Spector et al., 1998; Tjan et al., 2006; Pratte et al., 2013). Interestingly, emotional value of stimuli modulates the impact of the visual noise on brain activity. Namely, emotional stimuli are recognized at a lower threshold of noise (Reinders et al., 2005) and are perceived as less noisy (Markovic et al., 2014), which is linked to greater activity within the LOC and amygdala (Reinders et al., 2005; Todd et al., 2012b).

Several eye-tracking studies have shown that both the noise level and emotional content of an image affect fixations, which become longer as the clarity of an image decreases due to either high or low spatial filtering (Glaholt et al., 2013; Henderson et al., 2014) or other distortion methods preventing scene comprehension (Luke and Henderson, 2016). Emotional content also affects fixation number and duration. Bradley et al. (2011) established that participants make more fixations on negative and positive images than on neutral ones. Individual fixations executed while watching emotional images are also shorter (Bradley et al., 2011; Kaspar et al., 2013). A recently developed method of fMRI analysis allows the estimation of the relation between individual fixations and brain activity. FIBER analysis has so far been employed in a few experiments, whose results are not entirely consistent. Marsman et al. (2013) have shown that fixation duration correlates negatively with activity in the ventromedial visual cortex and early visual cortex. Contrarily, Henderson and Choi (2015) established that fixation duration is linked to activations within the primary visual cortex as well as the frontal gyrus and dorsolateral prefrontal cortex. Additionally, fixation duration was linked to deactivations in the cerebellum, brainstem, hippocampus, amygdala and paracentral lobule. These findings seem to be supported by the recent experiment by Marsman et al. (2016) in which occurrence of fixations was linked to an increase in activity within the early visual cortex accompanied by activations in the dorsal and ventral visual streams. Overall, it appears that fixation events are consistently linked to changes in activity within the early visual areas, however, the direction of this change remains unclear.

Since both decrease in visual noise and emotionality are related to the reduction of fixation duration, presumably their additive or interactive effect would influence brain correlates of fixations. It is possible that this modulation of fixation duration would be accompanied by changes in activity of the early visual areas, as reported by Marsman et al. (2013, 2016) and Henderson and Choi (2015). We expected fixation-related brain activity to be linearly correlated with the level of noise, and more pronounced in the case of negative images. Furthermore, based on evidence for more intense processing of emotional stimuli (for reviews, see Sabatinelli et al., 2011; Bradley et al., 2014; Lindquist et al., 2015; García-García et al., 2016), we expected that fixating emotional objects would be linked to greater fixation-related brain activity than fixating neutral objects. Lastly, we aimed to test whether the difference in brain activity accompanying fixations on objects and on the background would be larger for negative images over neutral ones.

In order to test our predictions, we used simultaneous fMRI and eye tracking recording, while presenting negative and neutral images with controlled object-background composition and varying signal-to-noise ratios. Then, we applied the FIBER method of fMRI data analysis, described by Marsman et al. (2012), in which fixation events are used as regressors. Marsman et al. (2012) have shown that despite being brief and frequently occurring, fixations yield themselves as valid event markers in an fMRI analysis. Additionally, we calculated the chance of fixating an object and average fixation duration in order to examine whether the modulating role of emotional content is also reflected in the eye-tracking data.

## Materials and Methods

### Participants

Twenty healthy participants (10 women), aged 19–29 years (*M* = 22.8) with normal or corrected-to-normal vision, no history of neurological disorders and free from any medical condition, were recruited by means of community advertisements on the Jagiellonian University Campus. A few days before the experiment, volunteers visited an fMRI facility where their ability to correctly perceive images through the VisualSystem goggles (NordicNeurolab, Bergen, Norway) was tested. Only those able to perceive a unitary percept through goggles were invited to participate in the experiment. Prior to the scanning session, participants signed an informed consent in accordance with the Declaration of Helsinki and an agreement to undergo an fMRI scanning procedure. The experimental procedure got the approval of the ethical committee of the Institute of Psychology at the Jagiellonian University.

### Experimental Material

A set of 25 negative (valence *M* = 2.62, SD = 0.47; arousal *M* = 6.35, SD = 0.58) and 25 neutral (valence *M* = 5.07, SD = 0.60; arousal *M* = 4.07, SD = 1.15) color images was selected from the International Affective Picture System (IAPS; Lang et al., 2008) and the Nencki Affective Picture System (NAPS; Marchewka et al., 2014) based on both valence and arousal ratings, which differed significantly between the image categories (for valence *t*_(48)_ = −16.07, *p* < 0.001; for arousal *t*_(48)_ = 8.85, *p* < 0.001). In both image databases, the valence and arousal were rated on scales 1–9, ranging from negative to positive valence, and from low to high arousal, respectively. We ensured that the mean size of the key object was the same for both negative and neutral images (10% and 11% of total image area, respectively; *t*_(48)_ = −1.0, *p* = 0.55) in order to match images in terms of picture composition.

The key objects within the images were determined in a procedure conducted prior to the fMRI experiment, in which participants (different to those taking part in the scanning procedure) were asked to circle key locations determining the valence of each image using a simple computer tool. The key objects were obtained by averaging the selections and by applying a threshold (a region encircled by at least 50% of the participants). A total of 602 images were rated in this procedure by 241 participants. Each participant rated 60–80 images. Each of the images selected to this fMRI study were rated on average by 29.6 participants (SD = 4.5). The procedure of determining key objects and the method of data analysis have been described in detail elsewhere (Pilarczyk and Kuniecki, 2014).

**Figure 1:**
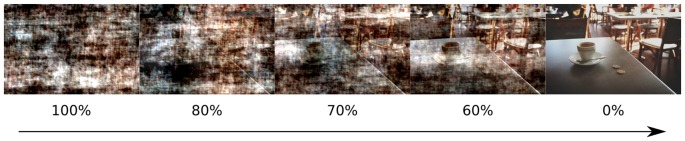
Example of images with superimposed noise, analogous to those presented in the experiment. Percentage of noise content is indicated below each image.

To manipulate the amount of visual information, pink noise was superimposed on the original images. Pink noise is obtained by replacing the phase in the Fourier spectrum of an original image with random values between 0 and 2 pi while keeping the amplitudes unchanged (Kayser et al., 2006). Pink noise was added to the original images in the following proportions: 0%, 60%, 70%, 80%, 100% (Figure 1), it was generated separately for each proportion and each image. All images were equated for luminance and contrast which were measured as a mean and standard deviation of L* component of the L*a*b* color space.

### Procedure

The experiment consisted of five scanning runs (each 10.8 min., 216 volumes). Each run was preceded by a nine-point calibration procedure. Images spanned 30 horizontal × 23 vertical degrees of the visual field. Stimuli were presented via the VisualSystem goggles. Images were shown for 5 s in fixed sequences from 100% to 0% of noise. Prior to each image, the fixation cross was presented for a time that randomly varied between 3 s and 6 s. Between the sequences the fixation cross was presented for a time window lasting 8 s on average, adjusted so that each sequence lasted precisely 60 s, which ensured that each run accommodated 10 sequences. The sequences were presented in a random order, ensuring, however, that in each run not more than seven sequences belonged to the same emotional category. In total, 50 unique sequences were presented during the entire experiment. To maintain participants’ attention throughout the runs, they were asked to classify one randomly selected image in each sequence as taken outdoors or indoors. The entire experimental procedure lasted approximately 60 min.

### Eye Movement Recording and Analysis

The ViewPoint infrared EyeTracker (Arrington Research, Scottsdale, AZ, USA) was used to measure eye movements at a 60 Hz sampling rate. A position of the right eye was recorded. A default nine-point calibration procedure was repeated at the beginning of each experimental block. Additionally, before each sequence a single point drift correction was performed. Fixations and saccades were detected using the default Arrington algorithm for the ViewPoint infrared EyeTracker (velocity below 26, 5°/s, drift less than 0.8° from the fixation point). Fixations on each image were analyzed to determine whether they fell on a key object, or outside the object, on the background. The duration of each fixation was also analyzed. The first fixation was defined as starting after the onset of an image.

To assess the chance of fixating an object, we calculated normalized fixation proportion (NFP), described in detail in Pilarczyk and Kuniecki (2014). This measure is similar in principle to other tests of classificator strength like the commonly used receiver operating curve (ROC) hence enabling the exclusion of such confounds as the size and position of an object—particularly its distance from the center of the screen—affecting fixation chance (Parkhurst et al., 2002; Tatler et al., 2005; Tatler, 2007). In brief, to calculate NFP the fraction of fixations that fall on the object while looking at the analyzed image (positive sample) is divided by the fraction of fixations made in this location in other images presented in the same noise and emotional condition (negative sample). If NFP equals one, the number of fixations on an object can be entirely explained by the object’s location and size. If it is larger, more fixations fall on an object than are predicted by chance. Fixation duration was analyzed using repeated measures ANOVA with factors of noise level (five levels), emotional category (negative and neutral) and fixation location (object, background). NFP was examined with repeated measures ANOVA including factors of noise (five levels) and emotional category (negative and neutral). In all cases where the sphericity assumption has been violated, the results are reported with H-F correction. Simple effects were investigated using Bonferroni correction.

### fMRI Data Acquisition

Magnetic resonance imaging (MRI) was performed using a 3T scanner (Magnetom Skyra, Siemens) with a 20-channel head coil. High-resolution, anatomical images were acquired using T1 MPRAGE sequence (sagittal slices; 1 × 1 × 1 mm^3^ voxel size; TR = 2200 ms, TE = 2.43 ms). Functional images were acquired using an EPI sequence; scan parameters were as follows: TR = 3000 ms, TE = 21 ms, flip angle = 90°, voxel size 2 × 2 × 2.5 mm^3^, FOV 192 × 192 mm^2^, GRAPPA acceleration factor 2, phase encoding A > P. Whole brain image (excluding cerebellum) was covered with 48 axial slices taken in an interleaved fashion. There were five functional runs; the acquisition time for each run was 10’48" (216 volumes). Due to magnetic saturation effects, the first four volumes (dummy scans) of each run were acquired and then discarded by the scanner.

The experimental task was presented through the VisualSystem goggles equipped with ViewPoint monocular eye-tracking cameras (infrared, 60 Hz) and responses were collected using fiber-optic response button grips (NordicNeuroLab, Bergen, Norway). Potential head motion was inhibited by using foam pads to stabilize head and arms and additionally by closely fitting the head-mounted goggles to participants’ eye-sockets.

### fMRI Data Analysis

Functional data were analyzed using FEAT FMRIB Expert Analysis Tool version 6.0^1^. The standard preprocessing steps included brain extraction using BET (Smith, 2002), slice timing correction, motion correction using MCFLIRT (Jenkinson et al., 2002), spatial smoothing with a Gaussian kernel of full-width at half-maximum of 5 mm and high pass temporal filtering with a 100 s cut-off. Next, whole brain general linear model (GLM) analysis was conducted for each of the five runs separately. For statistical inference, following Marsman et al. (2012), we used fixation onsets and durations to build our matrix for the GLM. For each emotional category, three fixation related regressors were specified: object fixation, background fixation, as well as all fixation onsets and durations parametrically modulated by the mean centered noise value. Additionally, onset of an image and diagnostic question were added as regressors of no interest. All regressors were convolved with a double-gamma hemodynamic response function (HRF). On a second level, each participant’s five runs were combined using fixed-effects. Group level analysis was conducted using a random-effects model with FLAME (Beckmann et al., 2003). Finally, parameter estimates were tested using RANDOMISE, an FSL tool for nonparametric inference based on permutation. We conducted 10K permutations and applied a threshold-free cluster enhancement (TFCE; Smith and Nichols, 2009) method with default parameters for identifying regions of continuous activation. The resulting statistical maps were thresholded at *p* < 0.05 (family-wise error).

## Results

### Eye-Tracking Results

#### Fixation Duration

**Figure 2:**
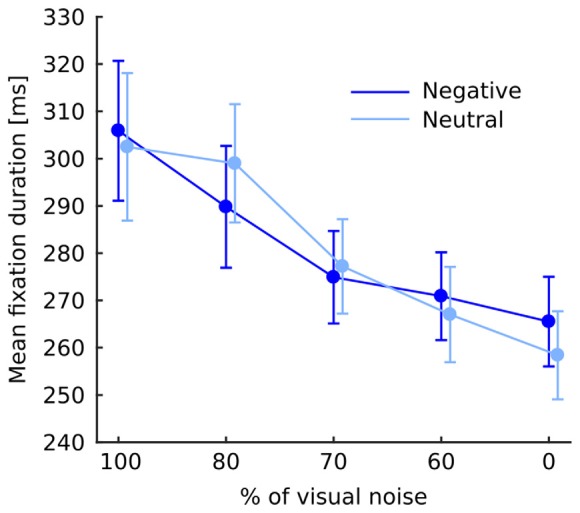
Mean fixation duration at each level of noise for negative and neutral images separately. Error bars represent standard error. The difference between negative and neutral condition is not significant and is shown for illustrative purposes only.

**Figure 3:**
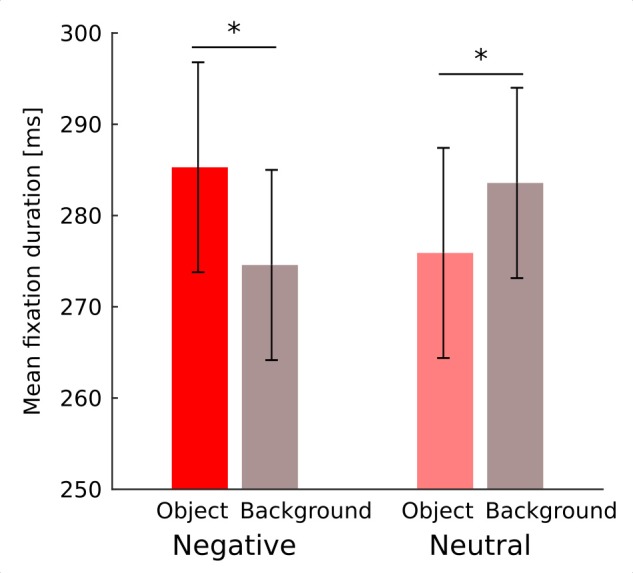
Mean duration of fixation falling on an object and on a background for negative and neutral images separately. Asterisks denote significant differences in pairwise comparisons. Error bars represent standard error.

Fixation duration was affected by noise level (*F*_(4,76)_ = 15.46; *p* < 0.001) showing a continuous decrease with diminishing noise as validated by significant linear trend (*F*_(1,19)_ = 25.34; *p* < 0.001). Interaction between noise level and emotional category did not reach significance (*F*_(4,76)_ = 1.1; *p* = 0.36; Figure 2). Interaction between emotional category and object-background was significant (*F*_(1,19)_ = 12.4; *p* = 0.002). Investigation of this effect with pairwise comparisons has revealed that in the negative condition fixations falling within an object were longer than those falling within the background (*p* = 0.022), while in the neutral condition the reverse effect emerged, specifically, fixations falling within an object were shorter than those falling within a background (*p* = 0.011; Figure 3). No other effects or interactions reached significance.

#### Normalized Fixation Proportion (NFP)

**Figure 4:**
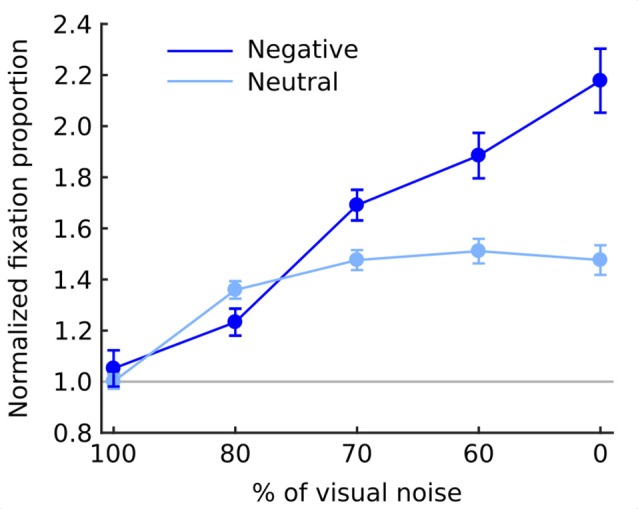
Normalized fixation proportion (NFP; chance of fixating an object) at each level of noise for negative and neutral images separately. Error bars represent standard error. Gray line represents the chance level.

The overall chance of fixating an object area was higher in the negative (1.61; SEM = 0.062) than in the neutral (1.36; SEM = 0.028) condition (*F*_(1,19)_ = 25.8; *p* < 0.001). Moreover, as the noise level decreased the chance of fixating an object steadily increased (*F*_(4,76)_ = 59.9; *p* < 0.001). This relationship was monotonic as indicated by a significant linear trend (*F*_(1,19)_ = 77.8; *p* < 0.001). Also interaction between emotional category and noise level was highly significant in both omnibus ANOVA (*F*_(4,76)_ = 29.4; *p* < 0.001) and a linear trend (*F*_(1,19)_ = 81.9; *p* < 0.001) with negative objects attracting fixations much stronger in decreasing noise levels than neutral objects (Figure 4).

### fMRI Results

**Figure 5:**
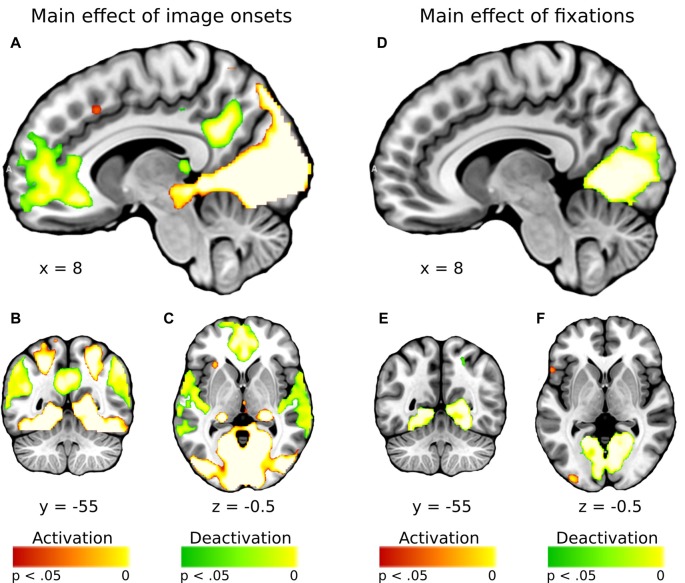
Whole-brain maps showing activations (in red) and deactivations (in green) linked to **(A–C)** main effect of image onsets and **(D–F)** main effect of fixations. T-statistical maps were corrected for multiple comparisons with threshold-free cluster enhancement (TFCE) at *p* < 0.05.

**Figure 6:**
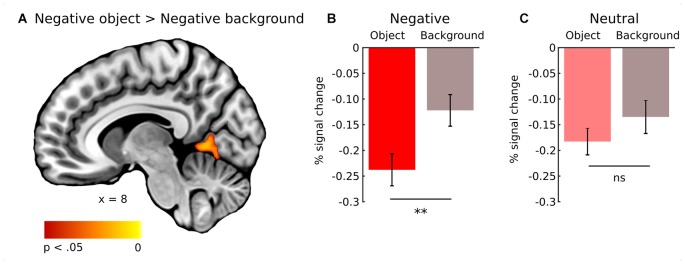
Whole-brain map showing contrast between activity linked to fixations on object and background of negative images. T-statistical maps were corrected for multiple comparisons with TFCE at *p* < 0.05. **(A)** Activations in the right lingual gyrus. Percent signal change in the right lingual gyrus for object and background of **(B)** negative and **(C)** neutral images. Significance of the pairwise comparisons are marked on the top of each graph; ns, not significant, **p < 0.01. Error bars represent standard error.

**Figure 7:**
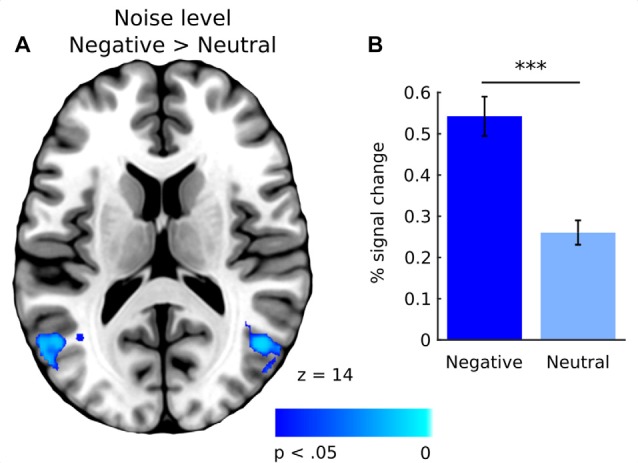
**(A)** Whole-brain map showing contrast between negative and neutral images for activations covarying with noise level. T-statistical maps were corrected for multiple comparisons using TFCE at *p* < 0.05. **(B)** Percent signal change in the lateral occipital cortex (LOC) for negative and neutral images. Significance of the pairwise comparisons are marked on the top of the graph; ns, not significant, ***p < 0.001. Error bars represent standard error.

In general, effect of fixations was linked to large deactivation within the bilateral lingual gyrus (Figure 5, Supplementary Table S1). Further, to separate the effects of fixations from image processing *per se*, we calculated effect of image onset to check whether image onsets and fixations impact brain activations distinctively. As expected, image onset yielded a very wide pattern of activations involving visual areas (including activation within lingual gyrus), as well as several frontal regions (Figure 5, Supplementary Table S2). In our opinion, this result shows that deactivation within the lingual gyrus is specifically related to fixations rather than image onset. Regarding contrasts pertaining to our specific hypotheses, in the negative condition fixations on objects were linked to significantly greater deactivations within the right lingual gyrus compared to background fixations (Figure 6, Table 1). In the neutral condition both object and background fixations resulted in similar deactivations within this region, yielding no significant contrast. Contrary to our predictions, there was no significant difference in fixation-related brain activity between fixating emotional and neutral objects. The emotional category modulated the effect of noise level on brain correlates of fixations. Specifically, in the negative condition the correlation between noise level and activity in the LOC linked to fixations was stronger than in the neutral condition (Figure 7, Table 1).

**Table 1:** Contrast between object and background fixations for negative images, and contrast between negative and neutral images for noise level (signal-to-noise ratio).

Brain region	Cluster size	Side	MNI coordinates			*t*	*p*
			*x*	*y*	*z*		
Object vs. Background fixations
Right lingual gyrus	543	R	8	−58	0	5.17	0.014
Intracalcarine cortex	59	R	14	−80	8	4.84	0.035
Lateral occipital cortex	51	R	28	−74	50	4.63	0.041
Signal-to-noise ratio
Lateral occipital cortex	694	R	54	−70	−2	6.46	0.003
Lateral occipital cortex	363	L	−54	−70	2	6.26	0.009

*p values are corrected for multiple comparisons with threshold-free cluster enhancement (TFCE). L, Left; R, Right. The cluster size is given in voxels*.

## Discussion

In the current study, we investigated whether the influence of low and high-level properties of an image on fixations and their brain correlates differ depending on the emotional category. We used FIBER method, that was recently developed by Marsman et al. (2012) to investigate fixations related brain activity. Although in Marsman et al. (2012) work the TR was 2 s, i.e., it was slightly shorter than the one used in our study, it was still an order of magnitude longer than the duration of an average fixation (200–300 ms). Furthermore, Yeşilyurt et al. (2008) have shown that it is possible to reliably detect BOLD change triggered by stimuli lasting as short as 5 ms with TR lasting 2 s. Moreover, short, sub-second ISI might increase the power of the design provided their randomization (Dale, 1999). Therefore, detection of BOLD changes related to events lasting around 200–300 ms with TR equaling 3 s should also be feasible. In fact, our analysis showed that fixations were primarily related to robust deactivations within the lingual gyrus. Furthermore, results indicated that emotional category affects brain activity linked to fixations on objects compared to the background as well as brain activity linked to fixations made in different levels of image clarity. These fMRI signal changes correspond to the eye-tracking data, whose temporal and spatial characteristics were affected by both emotional category and image properties.

Brain activity associated with fixations on an object differed from activity associated with fixations on the background, however only for negative images. In both emotional categories, all fixations were linked to deactivations in the lingual gyrus, however, in the case of negative images, they were significantly more pronounced for fixations that fell on the object. In the case of neutral images, there was no such difference. This result can be compared with the one reported by Ferri et al.’s (2013), who showed that brain activations were greater when participants were instructed to fixate an emotional object compared to fixating a non-emotional region. Those activations were, however, much more widespread, as they were located in the inferior occipital gyrus, insula, amygdala, inferior parietal lobe, postcentral gyrus and fusiform gyrus. The difference between the results reported by Ferri et al.’s (2013) and ours are not surprising due to major methodological differences between the two studies. Particularly, the FIBER analysis entails using fixation events as regressors and hence allows for evaluation of ongoing processes linked to individual fixations, rather than more global stimulus processing averaged across the entire presentation time. Further, we separated the latter from the fixation-related activity including image onset as the regressor of no interest. Moreover, in Ferri et al.’s (2013) procedure, participants were required to fixate a predetermined fragment of an image, that limited participants’ pattern of eye movements (compare eye-tracking data in free and constrained eye movement condition in Ferri et al.’s, 2013). In fact, the authors found number of differences in brain activations between focus vs. free-viewing condition (see Supplementary Tables S2, S4 in Ferri et al.’s, 2013).

The fact that only in the negative condition fixation-related activity in the right lingual gyrus differed between an object and the background can be interpreted with respect to research on semantic consistency. It has been shown that visual processing of an object depends on its semantic consistency with the context in which it is presented e.g., a polar bear presented in a kitchen or on an iceberg. In general, participants tend to focus more attention on an object than on the background, however, this tendency is significantly more robust for inconsistent images (Henderson et al., 1999; Võ and Henderson, 2009, 2011; Martens et al., 2011). In neutral natural scenes, objects do not seem to differ from the background in terms of their emotional value as much as in emotional scenes, in which emotional objects are often placed on a rather neutral background (Humphrey et al., 2012). In fact, Acunzo and Henderson (2011) claim that emotional objects resemble gist-inconsistent stimuli within an image, while the neutral ones resemble gist-consistent stimuli. Both gist-inconsistent and emotional objects draw attention more effectively, which can be a result of their greater informative value, possibly related to their behavioral and evolutionary relevance. Thus, in the case of neutral images, attention is distributed more evenly between an object and the background (Humphrey et al., 2012; Niu et al., 2012; Pilarczyk and Kuniecki, 2014). Our eye-tracking results fit into this line of reasoning. The probability of fixating an object was greater in the negative than in the neutral condition, which was also observed in our previous study (Pilarczyk and Kuniecki, 2014). Additionally, in the case of negative images, average duration of fixations falling on the objects was longer than those falling on the background. This tendency was reversed for neutral images. Taken together, these results demonstrate that, indeed, in emotional scenes, attention is captured and held more strongly by the object, which may account for the observed difference in fixation-related brain activity between an object and the background in the negative condition.

On the other hand, the results do not support our hypothesis regarding the difference in brain activity related to fixations on emotional objects compared to neutral ones. It seems that the intensity of processing of the “unit of information” extracted during a single fixation depends on the context in which it is presented rather than its absolute informative value, which, as we argue, should be greater in the case of negative objects than neutral ones. Furthermore, this pattern of results implies that considering fixations as “units of information” has some limitations, since brain activity associated with them seems to depend on the meaning of an entire scene, and hence probably on the preceding fixations. This hypothesis can be tested in future fMRI experiments, which would carefully control scanpaths and investigate the modulatory role of the previously executed fixations on brain correlates of the consecutive fixations. Future research might also explore the impact of the context in which information is presented on brain activity associated with fixations.

All observed effects of scene composition on fixation-related activity in the right lingual gyrus are deactivations. This result corresponds to the data obtained by Marsman et al. (2013) in the study, using artificial, non-emotional scenes. They found that fixation duration was related to deactivation in the lingual gyrus. One possible explanation of this effect is based on the study by Geng et al. (2009) showing that there is a link between increased activity in the lingual gyrus and saccade execution. We may hypothesize that deactivation of the lingual gyrus during fixations might be linked to delaying saccade execution and hence terminating the ongoing fixation. Furthermore, it seems plausible that the more a currently fixated object engages and holds attention, the greater the inhibition of gaze relocation, resulting in more pronounced deactivation of the lingual gyrus. In addition, the hypothesis that the content of a scene might influence the activity of the lingual gyrus related to eye movements is supported by Morris and McCarthy (2007), who established that among several brain regions activated by saccade execution, only activation in the ventral occipitotemporal cortex (VOTC), including the lingual gyrus, is modulated by the content of an image.

Indeed, our imaging and eye-tracking data seem to partly support proposed interpretation. Regarding brain activity, fixating an emotional object within negative images resulted in significantly greater deactivation of the right lingual gyrus than fixating non-emotional parts of the same image. This is reflected by the differences in fixation duration and location. In the case of negative images, fixations were longer when falling on objects as opposed to the background, which is reversed for neutral images. Further, the probability of fixating an emotional object was higher in the case of negative images than neutral ones. However, our results cannot be explained solely by differences in fixation duration. Specifically, if fixation duration was a major factor, we would expect greater deactivation within the lingual gyrus related to fixating the neutral background compared to the neutral object. This difference did not occur, indicating possible role of other factors influencing fixation-related brain activity within the region of the lingual gyrus.

The other modulatory effect of emotional content on fixation-related brain activity, found in our study, refers to scene clarity, which was positively correlated with activity in the LOC, the structure linked to object recognition (Grill-Spector et al., 2001). This correlation was significantly stronger in the case of fixations executed on negative images as opposed to neutral ones. We hypothesized that any possible interaction between emotional category and noise level in brain correlates of fixations would be related to fixation duration, which decreases in an emotional condition (Bradley et al., 2011; Kaspar et al., 2013) and with a declining noise level (Glaholt et al., 2013; Henderson et al., 2014). Our results, however, do not support this prediction. Although fixations became shorter as the level of noise decreased, the effect did not significantly differ between the emotional category conditions. Therefore, observed differences in brain correlates of fixations for negative and neutral images cannot be explained solely by differences in fixation duration.

Alternatively, this result may be an effect of either enhanced visual processing of emotional information or greater attentional deployment to emotional objects. It has been shown that visual noise affects processing in the higher visual areas, including the LOC, which are responsible for object and scene recognition (Grill-Spector et al., 1998; Tjan et al., 2006; Pratte et al., 2013). Interestingly, this effect is reduced in the case of emotional images (Todd et al., 2012b). It seems that our result is in line with these observations, indicating that such reduction is detectable also on the level of individual fixations. It is also possible that the increased LOC activity due to decreasing noise levels, which is stronger for negative images, might be attributed to the differences between attentional deployment during negative and neutral picture viewing. Our eye-tracking data directly supports this interpretation, as in the case of negative images, the chance of fixating an object rose as the noise level decreased, faster than in the case of neutral images. Such an impact of emotional content on attracting attentional focus between objects and background was also reported by Humphrey et al. (2012), Niu et al. (2012) and additionally including the influence of visual noise, by Pilarczyk and Kuniecki (2014).

Summing up, in our study emotional content changed the way that high and low-level features of an image affect fixation-related brain activity. In the case of emotional images, fixation-related brain activity in the right lingual gyrus differed between fixations on objects and on backgrounds, which was not the case for neutral images. We hypothesize that this difference might be driven by greater saccade inhibition when fixating an emotional object. This is supported by the fact that fixations on emotional objects were longer. Further, in the case of negative images, the relation between LOC activity and increasing image clarity was stronger, which, as we suggest, may be related to either enhanced processing of emotional stimuli or greater engagement of attention in emotional objects. Our eye-tracking results directly support the second interpretation, as the chance of fixating objects relative to the background was higher for emotional scenes than for neutral ones. Overall, it appears that the effects of high and low-level features of an image on fixation-related brain activity in the case of negative images are mediated by stronger engagement of attention by emotional objects.

## Author Contributions

MK and JP designed the study. MK, JP and AD conducted the study. MK, JP and KBW analyzed and interpreted the data. All authors wrote the manuscript.

## Conflict of Interest Statement

The authors declare that the research was conducted in the absence of any commercial or financial relationships that could be construed as a potential conflict of interest.

## Footnotes

^1^http://fsl.fmrib.ox.ac.uk/fsl/fslwiki/
