# Supplementary material for: Effects of Scene Properties and Emotional Valence on Brain Activations: A Fixation-Related fMRI Study
Source: Front Hum Neurosci. 2017 Aug 31;11:429. doi: 10.3389/fnhum.2017.00429 (PMC5583150; doi:10.3389/fnhum.2017.00429)
Supplement: Supplementary file 1 [file Table_1.docx]

| **Table 1. Fixation-related brain activity** | | | | | |  |
| --- | --- | --- | --- | --- | --- | --- |
| Brain region | Side | MNI coordinates | | | *t* value | *p* |
|  |  | x | y | z |  |  |
| Activations |  |  |  |  |  |  |
| Lateral Occipital Cortex | L | -48 | -62 | 28 | 4.82 | 0.016 |
| Occipital Pole | L | -34 | -94 | -4 | 7.57 | < 0.001 |
| Inferior Frontal Gyrus | L | -56 | 18 | 0 | 5.28 | 0.023 |
| Deactivations |  |  |  |  |  |  |
| Lingual Gyrus | R | 26 | -58 | -9 | -6.01 | < 0.001 |
| Lingual Gyrus | L | -20 | -55 | -2 | -7.36 | < 0.001 |
| *p values are corrected for multiple comparisons with TFCE. L, Left; R, Right.* | | | | | | |
