# Supplementary material for: Effects of Scene Properties and Emotional Valence on Brain Activations: A Fixation-Related fMRI Study
Source: Front Hum Neurosci. 2017 Aug 31;11:429. doi: 10.3389/fnhum.2017.00429 (PMC5583150; doi:10.3389/fnhum.2017.00429)
Supplement: Supplementary file 2 [file Table_2.docx]

| **Table 2. Brain activity linked to image onset** | | | | | |  |
| --- | --- | --- | --- | --- | --- | --- |
| Brain region | Side | MNI coordinates | | | *t* value | *p* |
|  |  | x | y | z |  |  |
| Activations |  |  |  |  |  |  |
| Lingual Gyrus | R | 12 | -78 | -10 | 10.42 | < 0.001 |
| Lingual Gyrus | L | -8 | -76 | -10 | 9.63 | < 0.001 |
| Lateral Occipital Cortex | R | 41 | -80 | 4 | 8.1 | < 0.001 |
| Lateral Occipital Cortex | L | -31 | -82 | 4 | 7.0 | < 0.001 |
| Temporal Fusiform Cortex | R | 34 | -26 | -32 | 12.1 | < 0.001 |
| Temporal Fusiform Cortex | L | -28 | -53 | -13 | 9.97 | < 0.001 |
| Occipital Fusiform Cortex | R | 15 | -77 | -12 | 10.73 | < 0.001 |
| Occipital Fusiform Cortex | L | -20 | -75 | -12 | 11.86 | < 0.001 |
| Precentral Gyrus | R | 46 | 10 | 26 | 6.46 | 0.003 |
| Superior Frontal Gyrus | L | -24 | -2 | 54 | 7.28 | 0.004 |
| Superior Frontal Gyrus | R | 27 | 2 | 91 | 5.81 | 0.003 |
| Inferior Frontal Gyrus | R | 42 | 9 | 24 | 5.82 | 0.004 |
| Inferior Frontal Gyrus | L | -44 | 10 | 24 | 7.13 | 0.002 |
| Paracingulate Gyrus | R | 2 | 12 | 50 | 5.56 | 0.013 |
| Supramarginal Gyrus | R | 42 | -34 | 38 | 4.67 | 0.29 |
| Frontal Orbital Cortex | L | -30 | 24 | -6 | 7.35 | 0.009 |
| Deactivations |  |  |  |  |  |  |
| Frontal Pole | - | 0 | 34 | -12 | -5.81 | < 0.001 |
| Planum Temporale | L | -46 | -12 | -4 | -5.16 | 0.002 |
| Planum Temporale | R | 43 | -13 | -4 | -5.40 | 0.004 |
| Precuneous Cortex | - | -6 | -56 | 24 | -6.63 | < 0.001 |
| Cingulate Gyrus Posterior Div | - | 0 | -24 | 44 | -6.21 | 0.009 |
| *p values are corrected for multiple comparisons with TFCE. L, Left; R, Right.* | | | | | | |
